# Enhancing Early Detection of Cognitive Decline in the Elderly through Ensemble of NLP Techniques: A Comparative Study Utilizing Large Language Models in Clinical Notes

**DOI:** 10.1101/2024.04.03.24305298

**Published:** 2024-04-05

**Authors:** Xinsong Du, John Novoa-Laurentiev, Joseph M. Plasek, Ya-Wen Chuang, Liqin Wang, Frank Chang, Surabhi Datta, Hunki Paek, Bin Lin, Qiang Wei, Xiaoyan Wang, Jingqi Wang, Hao Ding, Frank J. Manion, Jingcheng Du, Li Zhou

**Affiliations:** 1 Division of General Internal Medicine and Primary Care, Brigham and Women's Hospital, Boston, Massachusetts 02115; 2 Department of Medicine, Harvard Medical School, Boston, Massachusetts 02115; 3 Division of Nephrology, Taichung Veterans General Hospital, Taichung, Taiwan, 407219; 4 Intelligent Medical Objects, Rosemont, Illinois, 60018

**Keywords:** Cognitive Dysfunction, Natural Language Processing, Neurobehavioral Manifestations, Electronic Health Records, Early Diagnosis, Alzheimer Disease, Dementia

## Abstract

**Background:**

Early detection of cognitive decline in elderly individuals facilitates clinical trial enrollment and timely medical interventions. This study aims to apply, evaluate, and compare advanced natural language processing techniques for identifying signs of cognitive decline in clinical notes.

**Methods:**

This study, conducted at Mass General Brigham (MGB), Boston, MA, included clinical notes from the 4 years prior to initial mild cognitive impairment (MCI) diagnosis in 2019 for patients ≥ 50 years. Note sections regarding cognitive decline were labeled manually. A random sample of 4,949 note sections filtered with cognitive functions-related keywords were used for traditional AI model development, and 200 random subset were used for LLM and prompt development; another random sample of 1996 note sections without keyword filtering were used for testing. Prompt templates for large language models (LLM), Llama 2 on Amazon Web Service and GPT-4 on Microsoft Azure, were developed with multiple prompting approaches to select the optimal LLM-based method. Baseline comparisons were made with XGBoost and a hierarchical attention-based deep neural network model. An ensemble of the three models was then constructed using majority vote.

**Results:**

GPT-4 demonstrated superior accuracy and efficiency to Llama 2. The ensemble model outperformed individual models, achieving a precision of 90.3%, recall of 94.2%, and F1-score of 92.2%. Notably, the ensemble model demonstrated a marked improvement in precision (from a 70%-79% range to above 90%) compared to the best performing single model. Error analysis revealed 63 samples were wrongly predicted by at least one model; however, only 2 cases (3.2%) were mutual errors across all models, indicating diverse error profiles among them.

**Conclusion:**

Our findings indicate that LLMs and traditional models exhibit diverse error profiles. The ensemble of LLMs and locally trained machine learning models on EHR data was found to be complementary, enhancing performance and improving diagnostic accuracy.

## Summary

We found LLM, traditional machine learning, and deep learning had diverse error profiles on cognitive decline identification from clinical notes, and the ensemble of LLM, machine learning, and deep learning achieved state of the art performance.

## 1. Introduction

Alzheimer’s disease (AD) and related dementias (ADRD), the sixth leading cause of death in the US, affect 5.7 million Americans, with projections estimating an increase to 13 million by 2060.^1^ The progression of AD/ADRD significantly diminishes patient quality of life and places substantial emotional and financial strain on families, communities, and healthcare systems,^2^ with care costs anticipated to reach $1.1 trillion by 2050.^3^ Existing treatments, which only temporarily alleviate symptoms and decelerate progression,^4^ underscore the urgent need for breakthroughs in AD/ADRD therapy.^5^ Cognitive decline, characterized by noticeable deficits in cognitive functions beyond what might be expected from normal aging^6^ serves as a key early sign of AD/ADRD,^7^ emphasizing the necessity of timely detection for slowing disease advancement, facilitating involvement in clinical trials, and initiating early interventions.^8,9^

Electronic health records (EHRs) provide comprehensive, both real-time and historical, patient data, making them a critical resource for identifying cognitive decline. However, challenges arise with structured data like International Classification of Diseases (ICD) codes, which may lack the granularity to capture the spectrum of cognitive impairments, risking clinician under-reporting.^10^ Moreover, traditional diagnostic tools may fail to detect early stages of cognitive decline, increasing the risk of missed or incorrect diagnoses.^11^ The variability in screening practices further complicates early detection during standard medical evaluations.^12 13^ Additionally, constraints such as limited access to specialized care providers, including neurologists and geriatricians, exacerbate delays in diagnosis and treatment initiation.

Clinical notes within EHRs contain critical information on cognitive decline, detailing symptoms like memory loss, language difficulties, and impaired daily activities. Natural language processing (NLP) offers a powerful tool to identify these early signs of decline, which may not be coded in diagnoses. NLP facilitates the rapid and efficient analysis of large datasets, outpacing manual review. Researchers have investigated NLP techniques for detecting cognitive decline from clinical notes.^14^ For example, Penfold et al. utilized Python scripts to identify phrases indicative of cognitive function, predicting future mild cognitive impairment (MCI).^15^ Wang et al. created and validated a deep learning model to detect evidence of cognitive decline within EHR clinical notes.^10^ Nevertheless, no study has been done to investigate the effectiveness of large language model (LLM) on cognitive decline identification.

LLMs like GPT-4 and Llama 2, with their extensive pre-training on diverse text corpora, excel in understanding complex clinical narratives, offering advantages over traditional rule-based and machine learning approaches that are often trained from scratch on narrower clinical datasets. Their deep contextual comprehension enables the detection of subtle indicators of cognitive status within text.^16^ This research leverages LLMs within secure cloud computing environments for a pioneering exploration of EHR note analysis for cognitive decline detection. It also evaluates LLMs– effectiveness and interpretability against conventional machine learning methods and examines the synergy of LLMs with machine learning to enhance diagnostic accuracy. To the best of our knowledge, this initiative is the first of its kind to employ LLMs in such a capacity, representing a substantial innovation and contribution to the field.

## 2. Methods

### 2.1. Setting and Datasets

This study was conducted at Mass General Brigham (MGB), a large, integrated health care delivery system in Massachusetts. MGB has established secure HIPAA-compliant cloud environments for deploying and evaluating LLMs using actual EHR data. Two LLMs were included in this study: GPT-4^17^ via Microsoft Azure OpenAI Service Application Programming Interface (API) and Llama 2^18^ via an Amazon Elastic Compute Cloud (EC2) instance. Details about the two cloud environments were described in Supplementary Material Section 1 and Table S1. We set the temperature to 0 for LLMs to eliminate variability.

We used two datasets annotated from a previous study, which included sections of clinical notes over the four years leading up to the initial diagnosis of mild cognitive impairment (MCI) (ICD-10-CM code G31.84) in 2019 for patients aged 50 years or older.^10^ Because the positive case rate across any sections were low, a list of expert-curated keywords (Table 2) were used to screen sections that likely contain indications of cognitive decline. Dataset I included 4,949 sections filtered by keywords and were used for training two baseline models. For prompt development and LLM selection, we used a 200 random samples from Dataset I, and we name this dataset as Dataset I-S. Dataset II, a random sample of 1,996 sections not subjected to keyword filtering, were used for final testing.

The identification of cognitive decline encompasses various stages, ranging from subjective cognitive decline (SCD) to MCI to dementia, with a focus on the progressive nature of cognitive decline that is likely to correspond to or result in MCI. Cases considered transient (e.g., memory loss attributed to medication) or reversible were labeled as negative for cognitive decline.^10^

The study was approved by MGB Institutional Review Board with waiver of informed consent from study participants owing to secondary use of EHR data.

### 2.2. Prompt Development and LLM Selection

**Figure 1.**
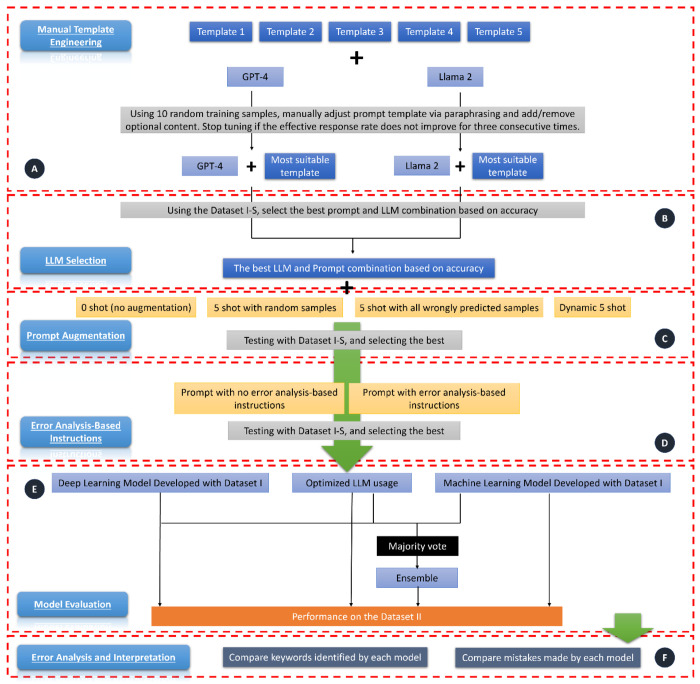
Study design overview. The workflow consists of five parts. A) Manual template engineering: we fed each prompt to GPT-4 and Llama 2 separately. If the effective response (i.e., the response answered the questions in the prompt) rate was not 100%, then manually adjust the template. If the effective response rate does not improve after tuning three times, then stop tuning and use the template leading to the best effective response rate. B) LLM selection: We used Dataset I-S and tested the accuracy of results produced by GPT-4 + its selected template and Llama 2 + its selected template. We used the best performing model and template version combination for following experiments. C) Prompt augmentation: we tested if five-shot prompting could improve the accuracy. D) Error analysis-based instructions: we tested if adding instructions following error analysis of GPT-4’s output on the Dataset I-S could improve the accuracy. E) Model evaluation: we evaluated the developed deep learning model, large language model, and machine learning model. We also tested the performance of ensemble model, which took the majority vote of the three models as the predictive label. F) Error analysis and interpretation: we exported keywords used by each model for the prediction, and compared the keywords with expert curated keywords, then we analyzed and compared errors made by each model.

As illustrated in Figure 1, to optimize LLM, we first selected the best prompt template for each model via manual template engineering^19^, then selected the best prompt template and model combination, finally, we tested if prompt augmentation^19^ with few shot and error analysis-based instructions^20^ could improve the model performance. Detailed steps for LLM development optimization are illustrated below.

Our task was to identify if a clinical note contains information that could identify the presence of cognitive decline, which is text classification. To complete the task using large language models, we developed requirements for prompts based on our needs, and then iteratively refined the prompts through team discussion. Since our problem belongs to clinical notes binary classification task, we need to measure the classification performance and interpret the result. Our requirements of the prompt include: 1) identify whether the clinical note has evidence showing cognitive decline or not; 2) output keywords that helped the LLM to make the judgment; and 3) ask the LLM to output its response to a JSON format, which makes it easier to use code to parse the result. Our internal discussion for the prompt improvement included: 1) to ask the LLM to output its reason for the judgment; 2) to include our definition of cognitive decline in the prompt. The temperature hyperparameter is used to control randomness and creativity, with 0 corresponding to a deterministic solution. During testing, we set the temperature to 0 to minimize randomness in response generation^20^. We categorized LLM responses into three categories as shown in Supplementary Table S2: 1) effective and parseable, 2) effective but not parseable, 3) not effective.

To develop the optimal prompt and model, we implement a structured approach as depicted in Figure 1. The process begins with prompt template engineering, where for each template, we manually tested 10 random samples from the training set to evaluate the percentage of effective responses. We then manually adjusted the prompt by paraphrasing or adding/removing optional content, such as definitions or requests for explanation of decisions, for each model. This tuning is stopped once there’s no improvement in the effective response rate after three consecutive attempts. Then, for the most effective prompt template identified for each LLM model, we used Dataset I-S to evaluate the percentage of parseable responses. We then assessed the accuracy, and chose the LLM that demonstrated superior accuracy. Next, we explored prompt augmentation to see if the inclusion of five examples (five-shot prompting) enhances performance. We used Template 6 in Supplementary Table S3 for the augmented prompt. Finally, we tested the effectiveness of error analysis-based instruction.

### 2.3. Baseline Models

We used XGBoost ^21^ as the machine learning model, and Convolutional Neural Network + Long-Short Term Memory with Hierarchical Attention as the deep learning model ^22,23^, since they achieved state-of-the-art performances according to our previous study^10^. XGBoost stands for Extreme Gradient Boosting and represents a scalable, distributed machine learning library based on gradient-boosted decision trees (GBDT). Renowned for its parallel tree boosting capabilities, it stands as the foremost choice for addressing regression, classification, and ranking challenges within the machine learning domain ^21^. A convolutional neural network (CNN) is a deep learning model specifically designed to process structured grid-like data, such as images or sequences ^24^. Long Short-Term Memory (LSTM) is a type of recurrent neural network (RNN) architecture designed to overcome the vanishing gradient problem in traditional RNNs and effectively capture long-range dependencies in sequential data ^25^. Connecting a LSTM neural network with an attention layer enhances interpretability by allowing the model to focus on relevant parts of the input sequence while making predictions^23^. For model parameter optimization, we used five-fold cross-validation on training data.

#### 2.3.1. Ensemble Model

To create the ensemble model, we take the majority vote of the LLM, deep learning model, and machine learning model as the label. The LLM is the best model selected from the above procedures. The deep learning model is the same as our previous study, which connects LSTM with attention. We employed XGBoost as our machine learning model since it was the best-performing machine learning model based on our previous study ^10^.

#### 2.3.2. Model Evaluation

Machine learning and deep learning models were trained and optimized using Dataset I. LLM and prompt were optimized using a subset (200 sectioned notes) of the 4,949 keyword filtered sections (Dataset I-S) due to budget limit. We tested each AI model on Dataset II, and reported precision, recall, and F1 score.

### 2.4. Error Analysis and Interpretation

Errors made by each model were analyzed and discussed among two biomedical informatician and a physician. For errors made by LLM, we also read the explanation output from the model. We also quantified unique and overlapping errors made by each model using a Venn Diagram. Regarding interpretation, we listed keywords that had a high frequency of appearance from LLM’s output (i.e., the number of appearances is higher than the average appearance time plus two standard deviations); keywords whose deep learning attention weights above the mean weights plus 2 standard deviations within individual sections; and keywords with an XGBoost information gain higher than the average value plus 2 standard deviations. We also listed expert curated keywords developed in our previous study as a reference^10^.

## 3. Results

Detailed dataset characteristics and model’s optimized hyperparameters are illustrated in the Supplementary Material. We highlighted model evaluation, interpretation, and error analysis below.

### 3.1. Performance evaluation

**Figure 2.**
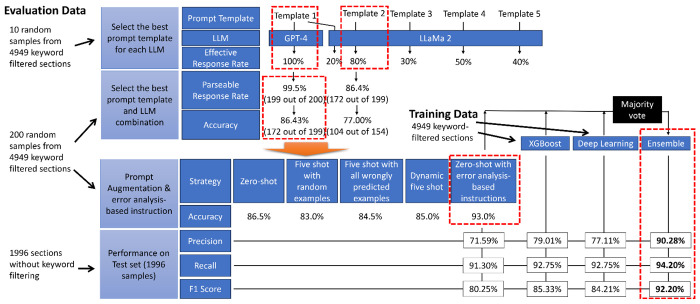
Evaluation results summary. During the prompt template selection, Template 1 was selected for GPT- 4 model due to an 100% effective response rate; Template 2 was selected for Llama 2 model since the effective response rate (80%) was not improved after tuning it for three times. Then, we compared the two combinations with 200 samples (Dataset I-S), and found GPT-4 + Template 1 had a much better accuracy (86.43%). Next, we found five shot prompting did not lead to a better performance, but adding error analysis-based instructions (i.e., GPT-4 + Template 7) could improve the accuracy to 93% on the Dataset I-S. Therefore, we selected using Template 7 as the prompt template, and GPT-4 as the LLM. On the test set, we evaluated the performances of developed machine learning model (XGBoost), developed deep learning model, and developed LLM. We found the XGBoost had a better performance: precision – 79.01%, recall – 92.75%, f1 score – 85.33%. Notably, after ensemble the three models using majority vote, we found the ensemble model had a much better performance: precision – 90.11% (11.1% improvement), recall – 94.20% (1.45% improvement), and f1 score – 92.20% (6.87% improvement).

As illustrated in Table 1 and Figure 2, we tested the Optimized LLM, XGBoost, and deep learning model on the test set. It turned out the LLM achieved a precision of 71.6%, a recall of 91.3%, and an F1 score of 80.3%. Optimized parameters for the deep learning model and XGBoost model are illustrated in Supplementary Table S8. The optimized XGBoost model achieved a precision of 79.0%, recall of 92.8%, and F1 score of 80.3%; the optimized deep learning model obtained a precision of 77.1%, recall of 92.8, and F1 score of 84.2%. Notably, an ensemble consisting of the LLM, deep learning model, and machine learning model boosted the performance (over 10% precision increase and around 7% F1-score improvement), which achieved a precision of 90.3%, recall of 94.2%, an F1 score of 92.2%.

### 3.2. Interpretation and Error Analysis

Table 2 contains keywords that have been identified through expert curation, exported by XGBoost, the deep learning model, and the LLM. These keywords cover a range of topics including memory-related issues such as recall and forgetfulness, cognitive impairments, and dementia with terms like dementia and Alzheimer, and evaluation and assessment methods, referencing tools like MoCA and MMSE. Comparing with traditional AI models and expert-selected keywords, GPT-4 highlighted specific treatment options, notably “Aricept” and “donepezil,” which are essential in managing dementia and Alzheimer’s disease. GPT-4 also identified specific diagnoses or conditions more explicitly than other models, with terms like “Mild Neurocognitive Disorder,” “major neurocognitive disorder,” and “vascular dementia.” Additionally, GPT-4 exported some keywords regarding emotional and psychological effects of cognitive disorders, such as “anxiety,” thus covering aspects sometimes overlooked by other models.

**Figure 3.**
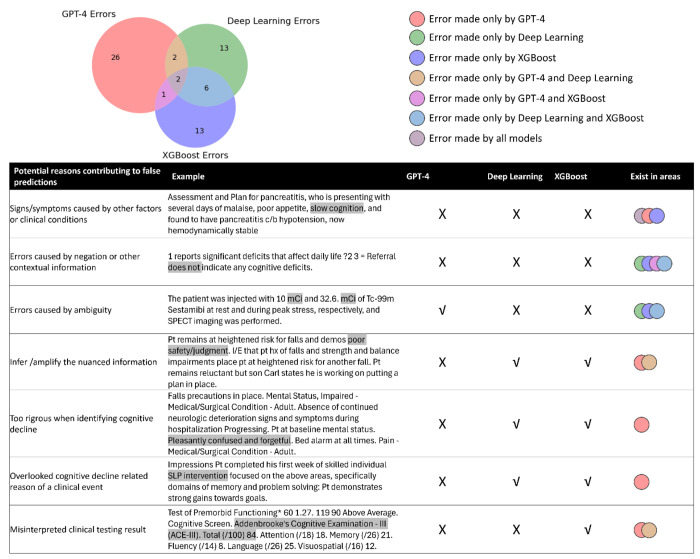
Venn Diagram highlighting unique and overlapping mistakes made by different models. √: correct prediction X: incorrect prediction. Some important findings include: 1) GPT-4 never predicts positive for cognitive decline if there is ambiguous information seemingly relevant to cognitive decline but not, while traditional AI approaches could make mistakes due to ambiguous terms; 2) GPT-4 could amplify the nuanced information traditional AI approaches, or be too rigorous, but traditional AI approaches do not have this issue; 3) all types of models could be misled by seemingly relevant symptoms/signs that normal people can have or very likely caused by signs/symptoms caused by other factors or clinical conditions.

An error analysis performed on LLM with test data is highlighted in Figure 3. We found all models could be misled by signs or symptoms as being caused by unrelated clinical conditions or be confounded by negations and other contextual details. Notably, GPT-4 stood out for its clarity in handling ambiguous terms, a common pitfall for traditional AI models. It shows an adeptness at inferring or emphasizing nuanced information that traditional AI methods typically overlook. However, GPT-4 could infer/amplify the nuanced information while traditional AI models did not make such mistakes. Additionally, GPT-4 was sometimes overly conservative, failing to identify cognitive decline despite compelling evidence. GPT-4 might also miss the underlying reasons for specific clinical events, such as visits or treatments related to cognitive decline. Both GPT-4 and deep learning models might misinterpret clinical testing results, marking an area for future improvement.

## 4. Discussion

Early detection of precursor stages of AD/ADRD, such as cognitive decline, becomes extremely important, as it can introduce treatment or intervention earlier to effectively reduce progression to AD/ADRD ^8,9^. In this study, we aimed to develop an NLP model for cognitive decline identification from clinical notes in EHR. We used data collected in our previous study for model development. Our study found that LLM did not outperform traditional AI for cognitive decline identification. This is because LLM was not trained specifically for a task, it is a more powerful tool than traditional AI models, and its output can have a wide range of responses, possibly giving ambiguous, inconsistent, and sometimes out-of-context answers^16^. We found although three types of models achieved similar performances, they made different types of errors. Notably, the ensemble of the three types of models boosted the performance (e.g., there was an over 10% precision improvement). Regarding interpretation, we found that LLM could identify keywords that were not identified by experts or the deep learning model, such as words related to medications. As illustrated in Supplementary Table S9, during error analysis, we found LLM could be very helpful to help physicians quickly identifying cognitive-decline related medications from a long medication report. Overall, our developed method can be a valuable tool for screening older patients to identify those with evidence of cognitive decline in clinical notes.

Many existing studies focused on using AI techniques to predict later stage of cognitive decline ^7,9,32–34^. A few studies were about NLP techniques for detecting cognitive decline. Moreira et al. (2018) ^35^ used unsupervised learning approach to cluster clinical notes and get pathological features, and then integrated structured EHR data for cognitive decline identification. They achieved a recall of 0.8 and an AUROC of 0.87. Wang et al. (2021) ^10^ developed a deep learning model (i.e., LSTM with attention model) for cognitive decline identification on clinical notes, and achieved a precision of 0.77 precision and a recall of 0.93. The study also found the attention-based deep learning model was able to identify useful keywords missed by experts. In 2022, Penfold et al. ^15^ developed a LASSO model using clinical notes and reported a precision of 0.70 and a recall of 0.17. More recently, Fouladvand et al. (2023) ^36^ integrated NLP techniques to identify cognitive decline using both structured and unstructured EHR data, and achieved an AUROC of 0.68 meanwhile ranked important predictors from both structured data and unstructured data. Compared with existing studies, our study achieved state-of-the-art performance (precision 0.90, recall 0.94, and f1 score 0.92) meanwhile identified keywords that were missed by experts and traditional AI models.

Our study has several strengths. Firstly, we set up private computational environments and tested two LLMs using cloud platforms. We noticed that Microsoft Azure GPT-4 outperformed AWS EC2 Llama 2 in terms of price, execution speed, and accuracy. However, as an open-source model, Llama 2 may have a better reproducibility ^37^ while GPT-4 may provide slightly different answers over time due to model updates from OpenAI ^38^. Secondly, we tested several prompting strategies, and found doing error analysis using some training data, and then asking LLM to avoid summarized common errors by revising the prompt can be an effective way to improve LLM’s performance. Additionally, we developed a prompt template for identifying cognitive decline from clinical note using LLM, and the template can also be used as a good reference when developing prompts for identifying other diseases. To the best of our knowledge, this is the first study employing LLM and unstructured EHR data for cognitive decline detection.

However, the results of this study should be considered along with some limitations. We used data from our previous study, which has several limitations including: 1) the data is from a single healthcare system, thus, external validation is still needed; 2) the data is retrospective and include notes written four years preceding the diagnose, but detecting cognitive decline beyond the 4-year window is still needed; 3) the data is unimodal, integrating other types of data (e.g., image, genomics) may further improve the performance. Additionally, considering the budget issue, the Llama 2 model used in our study is not the best one, which contains 70 billion parameters and requires more expensive hardware. Furthermore, our data is record-based and not patient based (I.e., longitudinal), thus, the developed model may not be able to distinguish well between reversable and progressive cognitive decline, since we do not know if the patient recovered later or not based only on a note section at one time point. An LLM-based early warning system of cognitive decline developed with longitudinal data would be a valuable future work.

## 5. Conclusion

This study is among the initial endeavors to utilize LLMs within HIPAA-compliant cloud environments, leveraging real EHR notes for the detection of cognitive decline. Our findings indicate that LLMs and traditional models exhibit diverse error profiles in comparison. The ensemble of LLMs and locally trained machine learning models on EHR data was found to be complementary, enhancing performance and improving diagnostic accuracy. Future work can leverage longitudinal and multimodal data to further improve performance.

## Supplementary Material

Supplement 1

## Figures and Tables

**Table 1.** Performance evaluation of the best LLM and prompting combination and machine learning models XGBoost is the best performing individual model, which achieved a precision of 79.01%, recall of 92.75%, and f1 score of 85.33%. The ensemble model boosted the best performance to precision – 90.28% (11.27% higher than XGBoost), recall – 94.20% (1.45% higher than XGBoost), f1 score – 92.20 (6.87% higher than XGBoost).

Model	Prompt Prefix Text Version	Precision	Recall	F1 Score	GPT-4 Execution Date
GPT4-8K	0 shot improved with error analysis	71.59%	91.30%	80.25%	01/03/2024
Deep Learning	NA	77.11%	92.75%	84.21%	-
XGBoost	NA	79.01%	92.75%	85.33%	-
Ensemble (vote of above 3 rows)	0 shot improved with error analysis	**90.28%**	**94.20%**	**92.20%**	01/03/2024

**Table 2.** Keywords contributing to identifying positive cognitive decline cases extracted from experts, deep learning model, and large language model. The table listed keywords that had a high frequency of appearance from LLM’s output (i.e., the number of appearances is higher than the average appearance time plus two standard deviations); keywords whose deep learning attention weights above the mean weights plus 2 standard deviations within individual sections; and keywords with an XGBoost information gain higher than the average value plus 2 standard deviations. We found keywords identified by AI models could significantly enrich the expert curated keyword set. Notably, only LLM identified keywords regarding medications for cognitive decline.

Model	Keywords
Expert Curated	Memory, delirium, dementia, psycha, neuroa, mental, alzheimer, confusa, mood, cognita, forgeta, agitate, moca, montreal, mmse, remember, difficult, recall, function, word, evaluata, score, drive, attention, mild, impairment, speech, question, disorientation, orientation, sleep, alter, exam, decline, worse, loss
XGBoost	Cognitive, memory, dementia, forgetful
Deep Learning	Memory, cognitive, dementia, impairment, neurocognitive, recall, decline, word, forgetful, cognition, MCI, executive, forgetfulness, alzheimer, remembering, MoCA, attention, deficits, recalling, forgetting, finding, words, visual, forgets, naming, difficulties, fluency, delay, alzheimers, retrieval, visuospatial, repetition, remember, hearing, cog, trails, language, FTD, frailty, encoding, developmental, delayed, behavioral, amnestic, phonemic, MMSE, falls, errors, attentional, speech, span, processing, neurodegenerative, lapses, HOH, deficit, correctly, auditory, years, spatial, solving, semantic, personality, perseveration, names, multidomain, moderately, linguistic, learning, LBD, items, insight, impaired, immediate, global, functioning, functional, expressive, died, cube, comprehension, clock, challenges, category, BNT, aphasia, amyloid, age, aforementioned, abstraction
GPT4-8K	dementia, cognitive impairment, memory loss, memory, mild cognitive impairment, neuropsychological evaluation, confusion, confused, Memory loss, forgetful, deficits, cognitive decline, Mild cognitive impairment, Cognitive impairment, Dementia, cognitive changes, Aricept, current level of cognitive functioning, memory impairment, Mild Neurocognitive Disorder, executive functioning, memory issues, attention, cognitive deficits, Memory impairment, MCI, neuropsychological tests, cognition, poor safety awareness, forgetfulness, Cognitive decline, cognitive difficulties, MOCA, memory problems, mild neurocognitive disorder, donepezil, neuropsychological testing, Impaired, memory concerns, major neurocognitive disorder, cognitive concerns, mild dementia, weakness, memory difficulties, working memory, altered mental status, concerns, MCI (mild cognitive impairment), neurodegenerative process, language, cognitive symptoms, neurocognitive status, Altered mental status, short term memory loss, cognitive issues, neurocognitive disorder, executive function, battery of neuropsychological tests, neuropsych testing, problem solving, verbal fluency, memory complaints, vascular dementia, word-finding difficulties, processing speed, cognitive-linguistic therapy, delirium, word finding difficulties, delayed recall, anxiety
