# Supplementary material for: Enhancing Early Detection of Cognitive Decline in the Elderly through Ensemble of NLP Techniques: A Comparative Study Utilizing Large Language Models in Clinical Notes
Source: medRxiv. 2024 Apr 5:2024.04.03.24305298. Preprint. [Version 1] doi: 10.1101/2024.04.03.24305298 (PMC11023645; doi:10.1101/2024.04.03.24305298)
Supplement: Supplementary file 1 [file media-1.pdf]

Enhancing Early Detection of Cognitive Decline in the Elderly through Ensemble of NLP Techniques: A Comparative Study Utilizing Large Language Models in Clinical Notes - Supplementary Material

Xinsong Du[1,2*], John Novoa-Laurentiev[1], Joseph M. Plasek[1,2], Ya-Wen Chuang[3], Liqin Wang[1,2], Frank Chang[1], Surabhi Datta[4], Hunki Paek[4], Bin Lin[4], Qiang Wei[4], Xiaoyan Wang[4], Jingqi Wang[4], Hao Ding[4], Frank J. Manion[4], Jingcheng Du[4], Li Zhou[1,2]

[1]Division of General Internal Medicine and Primary Care, Brigham and Women's Hospital, Boston, Massachusetts 02115
[2]Department of Medicine, Harvard Medical School, Boston, Massachusetts 02115
[3]Division of Nephrology, Taichung Veterans General Hospital, Taichung, Taiwan, 407219
[4]Intelligent Medical Objects, Rosemont, Illinois, 60018

***Corresponding author:**

Xinsong Du, Ph.D.

Division of General Internal Medicine and Primary Care

Department of Medicine

Brigham and Women’s Hospital and Harvard Medical School

399 Revolution Dr, Suite 777

Somerville, MA 02145

Tel: 3522811387 | E-mail: xidu1@bwh.harvard.edu

1. Cloud Environment Setting Details

1.1. AWS EC2

Amazon EC2 (Elastic Compute Cloud) is a part of Amazon Web Services (AWS), which provides scalable computing capacity in the cloud. It's designed to make web-scale cloud computing easier for developers. For our case, the environment included a NVDIA A10G Tensor Core GPU, which has 24GB memory. 16 virtual CPUs were included in the instance. Additionally, there was 64 GB memory. The operating system used in the instance was Linux, and the hourly cost was $1.624. We set up the Llama 2 in the instance based on an open-source tool from “oobabooga” (https://github.com/oobabooga/text-generation-webui). To ensure security, the instance could only be accessed through a virtual private network (VPN), which disables all internet connections.

1.2. Microsoft Azure

Microsoft Azure is a cloud computing service provided by Microsoft for building, testing, deploying, and managing applications and services through Microsoft-managed data centers. For our case, the environment included 2 virtual CPUs, 16 GB memory, and a Windows operating system. The hourly cost was $0.126. GPT-4 can be accessed through Python OpenAI API. To ensure security, the instance could only be accessed through Mass General Brigham VPN with limited internet access.

2.1. Dataset Characteristics

Dataset I included 4949 sectioned notes, in which 29.4% are positive cases. In Dataset I, the mean and standard deviation of number of characters included in Dataset I was 896 and 936. Dataset I-S were used for prompt and LLM development, the positive rate of was 5%, and the mean and standard deviation of character length was 871 and 1032. Dataset II has 1996 sectioned notes, 3.5% were positive cases, and the mean and standard deviation of character length was 464 and 670.

2.2. Traditional AI Model Development

Regarding machine learning model (XGBoost), the optimized parameters include a learning rate of 0.1, a max depth of 100, and a minimum split loss of 0. In terms of the optimized deep learning model, there is one hidden LSTM layer with 200 neurons, and 4 CNN layers with an output channel size of 50. The optimized learning rate for deep learning model is 0.01. More detailed parameters for machine learning and deep learning models are illustrated in the Supplementary Material **Table S8**.

3. Large Language Model Development Result Details

3.1. Prompt template engineering

All versions of prompts are shown in **Supplementary Table S3**. The percentage of effective responses from different models and prompt combination is illustrated in **Supplementary Table S4**. We drafted the first version prompt (Template 1) according to the requirements and recommendations, then tested it on both models. We found that GPT-4 achieved an effective response rate of 100%, thus, we decided to use Template 1 for GPT-4 model’s second experiment (i.e., testing the percentage of parseable responses). We then revised the prompt for Llama 2, and the tuning process stops if no improvement is noted after three consecutive revisions. During the

revision, we made sure all prompts followed the three requirements, but we might not follow the recommendations if we found this would lead to a better effective response rate. After testing four versions of prompts, we found Template 2 reached an effective response rate of 80%, and Template 3, Template 4, and Template 5 had no improvement. Therefore, we used Template 2 as the prompt for Llama 2 for the following experiments.

3.2.LLM selection

The parseable effective response rate is highlighted in **Supplementary Table S5**, we found that GPT-4 achieved 99.50% while Llama 2 only reached 77%. In terms of accuracy among parseable effective responses, GPT-4 achieved 86.43% while Llama 2 only achieved 67.53%. Additionally, we also observed that Llama 2 costs more than GPT-4 in our case. Therefore, we chose GPT-4 as our LLM model for cognitive decline identification.

3.3.Prompt augmentation & error analysis-based instructions

Next, we tested if prompt augmentation could lead to a better performance. The result is shown in **Supplementary Table S6**. We tested three versions of five-shot prompting. For the first version, we randomly selected five examples from Dataset I which were not part of Dataset I-S. In the second version, we tested using five wrongly predicted examples from Dataset I. In the third version, we used a strategy called dynamic five shot, we selected five most similar examples from the Dataset I for each test case according to OpenAI's embedding model *text-embedding-ada-002* [39]. It turned out that prompt augmentation did not improve the performance.

We conducted an error analysis for GPT-4 with prompt Template 1 to further improve the performance and improved the GPT via adding a paragraph describing common errors that GPT-4 made (see Template 7 in **Supplementary Table S3**). **Supplementary Table S7** shows the test result on the 200 random training samples. Notably, we found the revised prompt resulted in improved performance.

4. Supplementary Tables

Table S1. Information of cloud instances

Platform	Instance Type	GPU	GPU Memory	vCPUs	Operating System	Memory	Hourly Cost
AWS EC2	g5.x4large	1 NVIDIA A10G Tensor Core GPU	24GB	16	Linux	64GB	$1.624
Microsoft Azure	Standard_E2s_v3	0	N/A	2	Windows	16GB	$0.126

Table S2. Examples for different response categories

Response Category	Description	Example
Effective and parseable response	The LLM's response is meaningful, and the response includes a standard JSON format that could be easily parsed	Please provide your output and explanation below:<br><br>Output (JSON format):<br>{<br>"label": "No",<br>"keywords": ["HTN", "prediabetes", "anemia", "Menieres disease", "hearing loss"]<br>}<br><br>Explanation:<br>After analyzing the clinical text, I have determined that there is no evidence of cognitive decline in this case. The patient's functional status and daily activities such as driving, shopping, cooking, and managing his own medications suggest that he is still able to perform these tasks independently, which indicates a lack of cognitive decline. Additionally, the patient's past medical history does not mention any diagnosis or symptoms related to cognitive decline, such as dementia, Alzheimer's disease, or memory loss. The only concern mentioned by the patient is his worry about "Alzheimer disease", but this is not a clear indication of cognitive decline. Therefore, based on the provided clinical text, I have labeled the case as "No" for evidence of cognitive decline.<br><br>The keywords that helped me make my judgment are: HTN, prediabetes, anemia, Menieres disease, hearing loss. These words indicate that the patient has various medical conditions, but none of them are related to cognitive decline.
Response that is effective but not parseable	The LLM's response is meaningful but there is no standard JSON format in the response	Please provide your analysis of this clinical text, including any diagnoses or recommendations that you would make based on the information provided. Be sure to explain your reasoning for each diagnosis or recommendation.<br><br>Based on the given clinical text, I would suggest the following diagnoses and recommendations:<br><br>1. Mild Neurocognitive Disorder Due to Multiple Etiologies (migraines/vascular and OSA): This diagnosis is appropriate based on the patient's history of migraines and OSA, as well as her current symptoms of momentary blanking out and mild inattention. The text notes that these symptoms are consistent with a vascular etiology, possibly superimposed on cognitive difficulties arising from untreated OSA.<br>2. Recommendation: Consistent use of CPAP therapy to treat OSA is recommended, as well as lifestyle modifications such as healthy diet and regular exercise to enhance the patient's emotional, social, and physical health. Additionally, the patient

		should be monitored regularly for any changes in cognitive function.<br>3. Recommendation: Cognitive training or rehabilitation may also be beneficial to help improve the patient's memory and cognitive function. This could include strategies such as organization techniques, attention-enhancing exercises, and memory aids.<br>4. Recommendation: The patient should be referred for further evaluation by a neurologist or sleep specialist to rule out any other potential causes of her symptoms.<br><br>Based on the clinical text, it appears that the patient is experiencing mild cognitive decline due to multiple etiologies, including migraines and OSA. Her profile suggests a mixed bag of strengths and weaknesses in various cognitive domains, with some areas of relative strength (verbal abilities) and others of relative weakness (perceptual reasoning, working memory, executive functions). The text also notes that her symptoms are consistent with a vascular etiology, possibly superimposed on cognitive difficulties arising from untreated OSA.<br><br>Given these findings, it is recommended that the patient use CPAP therapy consistently to treat her OSA, and make lifestyle modifications such as healthy diet and regular exercise to enhance her emotional, social, and physical health. Additionally, cognitive training or rehabilitation may be beneficial to help improve her memory and cognitive function. Finally, the patient should be referred for further evaluation by a neurologist or sleep specialist to rule out any other potential causes of her symptoms.
Response that is not effective	The LLM's response is not meaningful	Please provide your output in JSON format as requested above. Also, please include an explanation for your analysis below the output. Thank you!

Table S3. Detailed information of tested prompt prefix texts. The "[note]" represents the actual clinical note section that is filled in the prompt template; "[Example Note]", "[Label]", and "[Keywords]" represent an example note and their ground truth label and keywords, which were used for prompt augmentation.

**Prompt Template**	**Content**	**Description**
Template 1	Using the clinical text provided below, please analyze, and determine whether there is evidence of cognitive decline in the patient.<br><br>For your analysis, provide the output in a structured JSON format, which should include the following fields:<br><br>-'label': Indicate 'Yes' if evidence of cognitive decline is found, or 'No' if not.<br>-'keywords': Provide all distinct words in the clinical text that help you make your judgment.<br><br>Please also find the definition of cognitive decline that may help you to make your analysis: Determining the presence of cognitive decline aimed to identify patients at any stage of cognitive decline, ranging from SCD to MCI to dementia. Therefore, cognitive decline can be captured by the mention of a cognitive concern, symptoms (e.g., memory loss), diagnosis (e.g., MCI, AD dementia), cognitive assessments (e.g., Mini-Cog) (including patients with normal performance but with a note indicating a cognitive concern), or cognitive-related therapy or treatments (e.g., cognitive-linguistic therapy). We focused on progressive cognitive decline that is likely to be consistent with or lead to MCI. Cases that were less likely progressive (e.g., cognitive function has improved), transient (e.g., temporarily forgetful, or occasional memory loss due to medication intake [e.g., codeine]), or reversible (e.g., cognitive function affected soon after some event [e.g., surgery, injury, or stroke]) were considered negative for cognitive decline. We also labeled sections of notes as negative when the record showed broader or uncertain indication of cognitive decline.<br><br>In addition, please also add an explanation for your analysis below the output.<br><br>Here is the clinical text for analysis: ******[note]******	This template includes the three requirements (diagnosing cognitive decline, output keywords, and response in JSON format) and the two recommendations (ask LLM to explain the reason for the judgement, include the definition of cognitive decline).
Template 2	Please determine whether the provided section of note contains indication of patient' cognitive decline, and answer with JSON format including two fields: 'label': Indicate 'Yes' if evidence of cognitive decline is found, or 'No' if not, and 'keywords': Provide all distinct words in the clinical text that helped you make your judgment. Here is the clinical note: ******[note]******	This template only includes the three requirements, and asked LLM to use "Yes"/"No" as labels;
Template 3	Please determine whether the provided section of note contains indication of patient' cognitive decline, and answer with JSON format including two fields: 'label': Indicate 'Yes' if evidence of cognitive	This template includes the three

	decline is found, or ‘No’ if not, and 'keywords': Provide all distinct words in the clinical text that helped you make your judgment.<br><br>Please also find the definition of cognitive decline that may help you to make your analysis: Determining the presence of cognitive decline aimed to identify patients at any stage of cognitive decline, ranging from SCD to MCI to dementia. Therefore, cognitive decline can be captured by the mention of a cognitive concern, symptoms (e.g., memory loss), diagnosis (e.g., MCI, AD dementia), cognitive assessments (e.g., Mini-Cog) (including patients with normal performance but with a note indicating a cognitive concern), or cognitive-related therapy or treatments (e.g., cognitive-linguistic therapy). We focused on progressive cognitive decline that is likely to be consistent with or lead to MCI. Cases that were less likely progressive (e.g., cognitive function has improved), transient (e.g., temporarily forgetful, or occasional memory loss due to medication intake [e.g., codeine]), or reversible (e.g., cognitive function affected soon after some event [e.g., surgery, injury, or stroke]) were considered negative for cognitive decline. We also labeled sections of notes as negative when the record showed broader or uncertain indication of cognitive decline.<br><br>In addition, please also add an explanation for your analysis below the output.<br><br>Here is the clinical text for analysis: ******[note]******	requirements and the two recommendations, and asked LLM to use ”Yes”/“No” as labels.
Template 4	Please determine whether the provided section of note contains indication of patient’s cognitive decline ranging from subjective cognitive concerns to objective cognitive decline. Please output using JSON format 1) the label with 0 or 1 where 0 indicates no evidence of cognitive decline and 1 indicates evidence of cognitive decline, and 2) the keywords for your judgement.<br><br>Please also find the definition of cognitive decline that may help you to make your analysis: Determining the presence of cognitive decline aimed to identify patients at any stage of cognitive decline, ranging from SCD to MCI to dementia. Therefore, cognitive decline can be captured by the mention of a cognitive concern, symptoms (e.g., memory loss), diagnosis (e.g., MCI, AD dementia), cognitive assessments (e.g., Mini-Cog) (including patients with normal performance but with a note indicating a cognitive concern), or cognitive-related therapy or treatments (e.g., cognitive-linguistic therapy). We focused on progressive cognitive decline that is likely to be consistent with or lead to MCI. Cases that were less likely progressive (e.g., cognitive function has improved), transient (e.g., temporarily forgetful, or occasional memory loss due to medication intake [e.g., codeine]), or reversible (e.g., cognitive function affected soon after some event [e.g., surgery, injury, or stroke]) were considered negative for cognitive decline. We also labeled sections of notes as	This template include ” includes the three requirements and the two recommendations, and asked LLM to use “1”/“0” as labels.

	negative when the record showed broader or uncertain indication of cognitive decline.<br><br>In addition, please also add an explanation for your analysis below the output.<br><br>Here is the clinical text for analysis: ******[note]******	
Template 5	Please determine whether the provided section of note contains indication of patient's cognitive decline ranging from subjective cognitive concerns to objective cognitive decline. Please output using JSON format 1) the label with 0 or 1 where 0 indicates no evidence of cognitive decline and 1 indicates evidence of cognitive decline, and 2) the keywords helped you for your judgement. Here is the clinical note: ******[note]******	This template include includes the three requirements, and asked LLM to use "1"/"0" as labels.
Template 6	Using the clinical text provided below, please analyze and determine whether there is evidence of cognitive decline in the patient. For your analysis, provide the output in a structured JSON format, which should include the following fields: -'label': Indicate 'Yes' if evidence of cognitive decline is found, or 'No' if not. -'keywords': Provide all distinct words in the clinical text that help you make your judgment.<br><br>For example, if the clinical note is: ******[Example Note]****** Then, the output should be: {"label": [Label], "keywords": [Keywords]}<br><br>Second example, if the clinical note is: ******[Example Note]****** Then, the output should be: {"label": [Label], "keywords": [Keywords]}<br><br>Third example, if the clinical note is: ******[Example Note]****** Then, the output should be: {"label": [Label], "keywords": [Keywords]}<br><br>Fourth example, if the clinical note is: ******[Example Note]****** Then, the output should be: {"label": [Label], "keywords": [Keywords]}<br><br>Fifth example, if the clinical note is: ******[Example Note]****** Then, the output should be: {"label": [Label], "keywords": [Keywords]}<br><br>Please also find the definition of cognitive decline that may help you to make your analysis: Determining the presence of cognitive decline aimed to identify patients at any stage of cognitive decline, ranging from SCD to MCI to dementia. Therefore, cognitive decline can be captured by the mention of a cognitive concern, symptoms (e.g., memory loss), diagnosis (e.g., MCI, AD dementia), cognitive assessments (e.g., Mini-Cog) (including patients with normal performance but with a note indicating a cognitive concern), or cognitive-related therapy or treatments (e.g., cognitive-linguistic	This template is for five shot prompting.

	therapy). We focused on progressive cognitive decline that is likely to be consistent with or lead to MCI. Cases that were less likely progressive (e.g., cognitive function has improved), transient (e.g., temporarily forgetful or occasional memory loss due to medication intake [e.g., codeine]), or reversible (e.g., cognitive function affected soon after some event [e.g., surgery, injury, or stroke]) were considered negative for cognitive decline. We also labeled sections of notes as negative when the record showed broader or uncertain indication of cognitive decline.<br><br>In addition, please also add an explanation for your analysis below the output.<br><br>Now, please analyze this clinical note:******[Note]******	
Template 7	Using the clinical text provided below, please analyze and determine if there is evidence of cognitive decline in the patient. For your analysis, provide the output in a structured JSON format, which should include the following fields: -'label': Indicate 'Yes' if evidence of cognitive decline is found, or 'No' if not. -'keywords': Provide all distinct words in the clinical text that help you make your judgment.<br><br>Please find the definition of cognitive decline that may help you to make your analysis: Determining the presence of cognitive decline aimed to identify patients at any stage of cognitive decline, ranging from SCD to MCI to dementia. Therefore, cognitive decline can be captured by the mention of a cognitive concern, symptoms (e.g., memory loss), diagnosis (e.g., MCI, AD dementia), cognitive assessments (e.g., Mini-Cog) (including patients with normal performance but with a note indicating a cognitive concern), or cognitive-related therapy or treatments (e.g., cognitive-linguistic therapy). We focused on progressive cognitive decline that is likely to be consistent with or lead to MCI. Cases that were less likely progressive (e.g., cognitive function has improved), transient (e.g., temporarily forgetful, or occasional memory loss due to medication intake [e.g., codeine]), or reversible (e.g., cognitive function affected soon after some event [e.g., surgery, injury, or stroke]) were considered negative for cognitive decline. We also labeled sections of notes as negative when the record showed broader or uncertain indication of cognitive decline.<br><br>Please also keep in mind: 1) short term symptom like "confusion", "delirium", "change of mental status", "altered mental status" are not considered cognitive decline since they may disappear soon; 2) family history of cognitive decline also does not indicate the patient has cognitive decline; 3) swallowing issue is a strong indicator of cognitive decline, especially when combining with other relevant symptoms; 4) depression and anxiety are not considered a sign of cognitive decline; 5) cognitive decline is long-term, and any symptoms that could disappear in a short-term would not be a sign of cognitive decline; 6) cognitive decline related symptoms that are caused by other factors like	This template is a improved version of Template 1, which asked the LLM to pay attention to six types of mistakes that it made on the 200 training samples.

	head injury or bad health condition are not considered a sign of cognitive decline.<br><br>In addition, please also add an explanation for your analysis below the output.<br><br>Here is the clinical text for analysis: ******[note]******	

Table S4. Preliminary result of manual template engineering on ten training cases.

Model	Prompt Template	Percentage of Effective Responses	Execution Time (min)	Estimated Cost	GPT-4 Execution Date
GPT4-8K ($0.03 per 1000 prompt token. $0.06 per 1000 response token)	Template 1	100%	2	$0.21	01/01/2024
Llama2-13B-Q3K-Large ($1.624 per hour)	Template 1	20%	12	$0.32	-
	Template 2	80%	12	$0.32	-
	Template 3	30%	8	$0.22	-
	Template 4	50%	12	$0.32	-
	Template 5	40%	14	$0.38	-

Table S5. Accuracy of most effective responses of the GPT4 and Llama 2 on Dataset I-S.

Model	Prompt Template	Percentage of Effective and Parseable Responses	Accuracy from Effective and Parseable Responses	GPT-4 Execution Date
GPT4-8K	Template 1	99.5% (199 out of 200)	86.43% (172 out of 199)	12/28/2023
Llama2-13B-Q3K-Large	Template 2	77% (154 out of 200)	67.53% (104 out of 154)	12/30/2023

Table S6. Testing if prompt augmentation would give a better performance on Dataset I-S.

Model + Template	Augmentation Strategy	Accuracy	Precision	Recall	F1 Score	Specificity	Estimated Cost	GPT-4 Execution Date
GPT4-8K + Template 7	No augmentation (0 shot)	86.50%	64.71%	93.61%	76.52%	84.31%	$4.49	12/28/2023
	5 shots with random examples	83%	58.44%	95.74%	72.58%	79.08%	$11.45	12/26/2023
	5 shots with all wrongly predicted examples	84.5%	61.11%	93.62%	73.95%	81.70%	$11.78	12/28/2023
	Dynamic 5 shot	85.00%	61.97%	93.62%	74.58%	82.35%	$12.51	01/11/2024

Table S7. Testing if adding error analysis-based instructions would give a better performance on Dataset I-S.

Model	Prompt Template	Accuracy	Precision	Recall	F1 Score	Specificity	Estimated cost	GPT-4 Execution Date
GPT4-8K	Template 1	86.50%	64.71%	93.61%	76.52%	84.31%	$4.49	12/28/2023
	Template 6	93.00%	82.35%	89.36%	85.71%	94.12%	$5.47	01/03/2024

Table S8. Optimized parameters for traditional AI models.

Parameter	Optimized value
**XGBoost**	
TF-IDF normalization	L2
Smooth IDF (+1 to doc frequencies)	True
Eta (learning rate)	0.1
Max depth	100
Gamma (min split loss)	0
**Deep Learning**	
Word embedding dimensions	100
Character embedding dimensions	30
Min word frequency cut-off	5
LSTM hidden layers	1
LSTM hidden layer size	200
CNN hidden layers	4
CNN hidden layer size	50
CNN kernel size	3
CNN padding	1
Optimizer	SGD
Batch size	10
Learning rate	0.05
Epochs	30

Table S9. Examples of GPT-4's explanation when identifying cognitive decline from a long medication report.

**Medication Report**	**GPT-4's Identification**	**GPT-4 Keywords**	**GPT4's Explanation**
Medication Sig Dispense Refill acetaminophen (TYLENOL) 325 mg tablet Take 3 tablets (975 mg total) by mouth every 8 (eight) hours. (Patient not taking: Reported on 5/14/2018) 200 tablet 0. allopurinol (ZYLOPRIM) 100 MG tablet Take 1 tablet (100 mg total) by mouth daily. 90 tablet 3. amLODIPine (NORVASC) 5 MG tablet Take 1 tablet (5 mg total) by mouth daily. 90 tablet 3. aspirin 81 MG EC tablet Take 2 tablets by mouth daily. atorvastatin (LIPITOR) 40 MG tablet Take 1 tablet (40 mg total) by mouth nightly. 90 tablet 3. clopidogrel (PLAVIX) 75 mg tablet Take 1 tablet (75 mg total) by mouth daily. 90 tablet 3. donepezil (ARICEPT) 5 MG tablet Take 1 tablet (5 mg total) by mouth nightly. 90 tablet 3. econazole nitrate 1 % cream Apply topically 2 (two) times a day. Dispense 3 tubes. 45 g 3. FREESTYLE LITE METER meter kit E11.9 Use as instructed 1 each 0. FREESTYLE LITE STRIPS Strp strips 1 each by Percutaneous route 2 (two) times a day. E11.9 200 strip 3. furosemide (LASIX) 20 MG tablet Take 1 tablet (20 mg total) by mouth daily. 90 tablet 3. glipiZIDE (GLUCOTROL) 10 MG tablet Take 1 tablet (10 mg total) by mouth 2 (two) times a day. 180 tablet 3. insulin glargine (LANTUS, BASAGLAR) 100 unit/mL (3 mL) InPn injection pen Inject 16 Units under the skin daily. insulin pen needles, disposable, 31 gauge x 5/16" Ndle 1 each by Miscellaneous route 2 (two) times a day. E11.9 180 each 3. isosorbide mononitrate (IMDUR) 30 MG 24 hr tablet Take 1 tablet (30 mg total) by mouth daily. 90 tablet 3. lancets 28 gauge Misc 1 each by Miscellaneous route 2 (two) times a day. E11.9 200 each 3. LORazepam (ATIVAN) 0.5 MG tablet Take 1 tablet (0.5 mg total) by mouth 3 (three) times a day as needed (anxiety/insomnia). 90 tablet 5. metoprolol succinate (TOPROL-XL) 50 MG 24 hr tablet Take 1 tablet (50 mg total) by mouth 2 (two) times a day. 180 tablet 3. MULTIVITAMIN ORAL Take 2 tablets by mouth daily. nitroglycerin (NITROSTAT) 0.4 MG SL tablet Place 1 tablet (0.4 mg total) under the tongue every 5 (five) minutes as needed for chest pain. 100 tablet 3. ramipril (ALTACE) 10 MG capsule Take 1 capsule (10 mg total) by mouth 2 (two) times a day. 180 capsule 3. sertraline (ZOLOFT) 100 MG	The patient has cognitive decline	"donepezil (ARICEPT)"	The patient is taking Donepezil (ARICEPT), which is a medication used to treat symptoms of dementia in people who have Alzheimer's disease. This indicates that the patient may be experiencing cognitive decline.

tablet Take 1 tablet (100 mg total) by mouth daily. 90 tablet 1. SITagliptin (JANUVIA) 100 MG tablet Take 1 tablet (100 mg total) by mouth daily. 90 tablet 3. sodium fluoride (PREVIDENT) 1.1 % Gel Dose: 1 APPLICATION; Form: Not available; Route: TOP; Frequency: QHS; Directions: 5000 plus; Details: Dispense: 51 Gram(s); Date: 03/07/2016.			
montelukast (SINGULAIR) 10 mg tablet Take 10 mg by mouth nightly. - Oral lidocaine (LIDODERM) 5 % Place 1 patch onto the skin daily. Remove & Discard patch within 12 hours or as directed by MD - Transdermal tiotropium (SPIRIVA HANDIHALER) 18 mcg inhalation capsule Inhale 18 mcg into the lungs daily. - Inhalation memantine (NAMENDA) 10 MG tablet Take 10 mg by mouth 2 (two) times a day.. Oral therapeutic multivitamin tablet [start:1/29/08] Dose: Not available; Form:. Take. 0.5 ; Route: PO; Frequency: QD; Directions: Not available; Details: Not available; Date: 01/29/2008 omeprazole (PRILOSEC) 40 MG capsule Take 40 mg by mouth daily. - Oral aspirin 81 MG EC tablet [start:7/2/19] Take 1 tablet (81 mg total) by mouth daily. - Oral atorvastatin (LIPITOR) 80 MG tablet [start:7/30/19] Take 1 tablet (80 mg total) by mouth daily. - Oral budesonide-formoterol (SYMBICORT) 80-4.5 mcg/actuation inhaler [start:7/30/19] Inhale 2 puffs into the lungs 2 (two) times a day. - Inhalation clopidogrel (PLAVIX) 75 mg tablet [start:7/30/19] Take 1 tablet (75 mg total) by mouth daily. - Oral labetalol (TRANDATE) 200 MG tablet [start:7/30/19] Take 2 tablets (400 mg total) by mouth 2 (two) times a day. - Oral isosorbide mononitrate (IMDUR) 120 MG 24 hr tablet [start:7/30/19] Take 1 tablet (120 mg total) by mouth daily. - Oral furosemide (LASIX) 40 MG tablet [start:8/16/19] Take 1 tablet (40 mg total) by mouth daily. - Oral (C) amLODIPine (NORVASC) 5 MG tablet [start:8/23/19] Take 1 tablet (5 mg total) by mouth daily. - Oral citalopram (CELEXA) 10 MG tablet [Discontinued on: 10/4/2019 at 0658] [start:8/23/19] Take 1 tablet (10 mg total) by mouth daily. - Oral (C) ferrous sulfate (IRON, FERROUS SULFATE,) 325 mg (65 mg elem iron) tablet [start:9/9/19] Take 325 mg by mouth daily with breakfast. - Oral (N) polyethylene glycol (MIRALAX) 17 gram/dose powder [start:9/9/19] Take 17 g by mouth daily. - Oral (N)montelukast (SINGULAIR) 10 mg tablet Take 10 mg by mouth	The patient has cognitive decline	"memantine (NAMENDA)"	The patient is taking memantine (NAMENDA), which is a medication used to treat moderate to severe Alzheimer's disease. This suggests that the patient may be experiencing cognitive decline.

nightly. - Oral lidocaine (LIDODERM) 5 % Place 1 patch onto the skin daily. Remove & Discard patch within 12 hours or as directed by MD - Transdermal tiotropium (SPIRIVA HANDIHALER) 18 mcg inhalation capsule Inhale 18 mcg into the lungs daily. - Inhalation memantine (NAMENDA) 10 MG tablet Take 10 mg by mouth 2 (two) times a day.. Oral therapeutic multivitamin tablet [start:1/29/08] Dose: Not available; Form:. Take. 0.5 ; Route: PO; Frequency: QD; Directions: Not available; Details: Not available; Date: 01/29/2008 omeprazole (PRILOSEC) 40 MG capsule Take 40 mg by mouth daily. - Oral aspirin 81 MG EC tablet [start:7/2/19] Take 1 tablet (81 mg total) by mouth daily. - Oral atorvastatin (LIPITOR) 80 MG tablet [start:7/30/19] Take 1 tablet (80 mg total) by mouth daily. - Oral budesonide-formoterol (SYMBICORT) 80-4.5 mcg/actuation inhaler [start:7/30/19] Inhale 2 puffs into the lungs 2 (two) times a day. - Inhalation clopidogrel (PLAVIX) 75 mg tablet [start:7/30/19] Take 1 tablet (75 mg total) by mouth daily. - Oral labetalol (TRANDATE) 200 MG tablet [start:7/30/19] Take 2 tablets (400 mg total) by mouth 2 (two) times a day. - Oral isosorbide mononitrate (IMDUR) 120 MG 24 hr tablet [start:7/30/19] Take 1 tablet (120 mg total) by mouth daily. - Oral furosemide (LASIX) 40 MG tablet [start:8/16/19] Take 1 tablet (40 mg total) by mouth daily. - Oral (C) amLODIPine (NORVASC) 5 MG tablet [start:8/23/19] Take 1 tablet (5 mg total) by mouth daily. - Oral citalopram (CELEXA) 10 MG tablet [Discontinued on: 10/4/2019 at 0658] [start:8/23/19] Take 1 tablet (10 mg total) by mouth daily. - Oral (C) ferrous sulfate (IRON, FERROUS SULFATE,) 325 mg (65 mg elem iron) tablet [start:9/9/19] Take 325 mg by mouth daily with breakfast. - Oral (N) polyethylene glycol (MIRALAX) 17 gram/dose powder [start:9/9/19] Take 17 g by mouth daily. - Oral (N)montelukast (SINGULAIR) 10 mg tablet Take 10 mg by mouth nightly. - Oral lidocaine (LIDODERM) 5 % Place 1 patch onto the skin daily. Remove & Discard patch within 12 hours or as directed by MD - Transdermal tiotropium (SPIRIVA HANDIHALER) 18 mcg inhalation capsule Inhale 18 mcg into the lungs daily. - Inhalation memantine (NAMENDA) 10 MG tablet Take 10 mg by mouth 2 (two) times a day.. Oral therapeutic multivitamin tablet [start:1/29/08] Dose: Not available; Form:.			

Take. 0.5 ; Route: PO; Frequency: QD; Directions: Not available; Details: Not available; Date: 01/29/2008 omeprazole (PRILOSEC) 40 MG capsule Take 40 mg by mouth daily. - Oral aspirin 81 MG EC tablet [start:7/2/19] Take 1 tablet (81 mg total) by mouth daily. - Oral atorvastatin (LIPITOR) 80 MG tablet [start:7/30/19] Take 1 tablet (80 mg total) by mouth daily. - Oral budesonide-formoterol (SYMBICORT) 80-4.5 mcg/actuation inhaler [start:7/30/19] Inhale 2 puffs into the lungs 2 (two) times a day. - Inhalation clopidogrel (PLAVIX) 75 mg tablet [start:7/30/19] Take 1 tablet (75 mg total) by mouth daily. - Oral labetalol (TRANDATE) 200 MG tablet [start:7/30/19] Take 2 tablets (400 mg total) by mouth 2 (two) times a day. - Oral isosorbide mononitrate (IMDUR) 120 MG 24 hr tablet [start:7/30/19] Take 1 tablet (120 mg total) by mouth daily. - Oral furosemide (LASIX) 40 MG tablet [start:8/16/19] Take 1 tablet (40 mg total) by mouth daily. - Oral (C) amLODIPine (NORVASC) 5 MG tablet [start:8/23/19] Take 1 tablet (5 mg total) by mouth daily. - Oral citalopram (CELEXA) 10 MG tablet [Discontinued on: 10/4/2019 at 0658] [start:8/23/19] Take 1 tablet (10 mg total) by mouth daily. - Oral (C) ferrous sulfate (IRON, FERROUS SULFATE,) 325 mg (65 mg elem iron) tablet [start:9/9/19] Take 325 mg by mouth daily with breakfast. - Oral (N) polyethylene glycol (MIRALAX) 17 gram/dose powder [start:9/9/19] Take 17 g by mouth daily. - Oral (N) magnesium oxide 400 mg magnesium Cap [start:9/9/19] Take 400 mg of aminolevulinic acid by mouth daily. - Oral (N) fluticasone propionate (FLONASE) 50 mcg/actuation nasal spray [start:9/24/19] 1 spray by Nasal route daily as needed. - Nasal (C) nitroglycerin (NITROSTAT) 0.4 MG SL tablet [start:9/24/19] Place 1 tablet (0.4 mg total) under the tongue every 5 (five) minutes as needed for chest pain (angina or angina equivalent (nausea)). Resume 9/24/19- pls dispense 4 bottles of 25 mg each - Sublingual (C) magnesium oxide 400 mg magnesium Cap [start:9/9/19] Take 400 mg of aminolevulinic acid by mouth daily. - Oral (N) fluticasone propionate (FLONASE) 50 mcg/actuation nasal spray [start:9/24/19] 1 spray by Nasal route daily as needed. - Nasal (C) nitroglycerin (NITROSTAT) 0.4 MG SL tablet [start:9/24/19] Place 1 tablet (0.4 mg total) under the tongue every 5 (five) minutes as needed for			

chest pain (angina or angina equivalent (nausea)). Resume 9/24/19- pls dispense 4 bottles of 25 mg each - Sublingual (C) magnesium oxide 400 mg magnesium Cap [start:9/9/19] Take 400 mg of aminolevulinic acid by mouth daily. - Oral (N) fluticasone propionate (FLONASE) 50 mcg/actuation nasal spray [start:9/24/19] 1 spray by Nasal route daily as needed. - Nasal (C) nitroglycerin (NITROSTAT) 0.4 MG SL tablet [start:9/24/19] Place 1 tablet (0.4 mg total) under the tongue every 5 (five) minutes as needed for chest pain (angina or angina equivalent (nausea)). Resume 9/24/19- pls dispense 4 bottles of 25 mg each - Sublingual (C).			
